# Primary biliary cholangitis: molecular pathogenesis perspectives and therapeutic potential of natural products

**DOI:** 10.3389/fimmu.2023.1164202

**Published:** 2023-06-30

**Authors:** Yanling Zhao, Shizhang Wei, Lisheng Chen, Xuelin Zhou, Xiao Ma

**Affiliations:** ^1^ Department of Pharmacy, Chinese People's Liberation Army (PLA) General Hospital, Beijing, China; ^2^ Department of Anatomy, Histology and Embryology, School of Basic Medical Sciences, Peking University, Beijing, China; ^3^ College of Pharmacy, Chengdu University of Traditional Chinese Medicine, Chengdu, China; ^4^ Department of Pharmacology, School of Basic Medical Sciences, Capital Medical University, Beijing, China

**Keywords:** PBC, pathological mechanism, treatment options, drug discovery, natural products

## Abstract

Primary biliary cirrhosis (PBC) is a chronic cholestatic immune liver disease characterized by persistent cholestasis, interlobular bile duct damage, portal inflammation, liver fibrosis, eventual cirrhosis, and death. Existing clinical and animal studies have made a good progress in bile acid metabolism, intestinal flora disorder inflammatory response, bile duct cell damage, and autoimmune response mechanisms. However, the pathogenesis of PBC has not been clearly elucidated. We focus on the pathological mechanism and new drug research and development of PBC in clinical and laboratory in the recent 20 years, to discuss the latest understanding of the pathological mechanism, treatment options, and drug discovery of PBC. Current clinical treatment mode and symptomatic drug support obviously cannot meet the urgent demand of patients with PBC, especially for the patients who do not respond to the current treatment drugs. New treatment methods are urgently needed. Drug candidates targeting reported targets or signals of PBC are emerging, albeit with some success and some failure. Single-target drugs cannot achieve ideal clinical efficacy. Multitarget drugs are the trend of future research and development of PBC drugs.

## 1 Introduction

Primary biliary cirrhosis (PBC) is clinically a type of autoimmune cholestatic liver disease characterized by serum autoantibody antimitochondrial antibodies (AMAs) (1, 2), chronic progressive cholestasis, interlobular bile duct damage, liver inflammation, eventual cirrhosis, and death (3, 4). Studies have found that the incidence and prevalence of PBC have increased globally in the past few decades (5, 6), especially among women (7). About 90% of patients with PBC have developed AMA against PDC-E2 (8). Importantly, AMA is still not detected in the serum of 5% of patients with PBC. However, liver tissues of patients with AMA-negative PBC show anti-nuclear antibodies (9, 10). Many patients with PBC suffer from complications, such as sicca complex (34%) (11), osteoporosis (20%–40%) (12), and hyperlipidemia (75%–95%) (13).

At present, the research on serum markers in PBC has made great breakthroughs. Some new highly sensitive PBC autoantigens (14–16) and PBC biomarkers with extremely high accuracy (17, 18) have been discovered. With the introduction of genome-wide association analysis (GWAS), some key gene loci have been discovered (19–21). It gives us deeper insight into the pathogenesis of PBC. However, the key pathological mechanism of PBC has not yet been clarified. Moreover, effective medicines for PBC still cannot meet clinical needs. Ursodesoxycholic acid (UDCA) can improve approximately two of the three of patients with PBC, but there are still approximately 30% of patients with PBC who are not responsive to UDCA (22). Encouragingly, obeticholic acid (OCA) can treat patients with UDCA-unresponsive PBC. However, it can aggravate the itching symptoms of patients with PBC (23). The only option for advanced PBC is liver transplantation (24). Although the pathogenesis of PBC has a great relationship with the immune disorder, the classic immunosuppressive agents are not ideal for PBC (25–27). Therefore, we hope that, by searching for the PBC pathogenesis, the key pathological mechanism of PBC can be dug out and provide evidence for targeted therapy of PBC in the future.

## 2 “Bile acid–intestinal flora–bile acid” signal axis in the progress of PBC

There are 10^13^–10^14^ microbial cells in the human intestine and more than 1,000 species of bacteria (28–30). The microbiota composition changes may cause increased intestinal permeability and bacterial translocation, eventually leading to chronic liver inflammation and fibrosis (31). Bile acids (BAs; 5%) in the colon are transformed into secondary BAs by intestinal bacteria (32). BAs can regulate the composition of intestinal microbes (32). In the early stage of PBC, the reduction of several potentially beneficial microbiota and the enrichment of opportunistic pathogens were observed (30, 33). Some flora can accurately distinguish patients with PBC from normal people (34).

**Figure 1:**
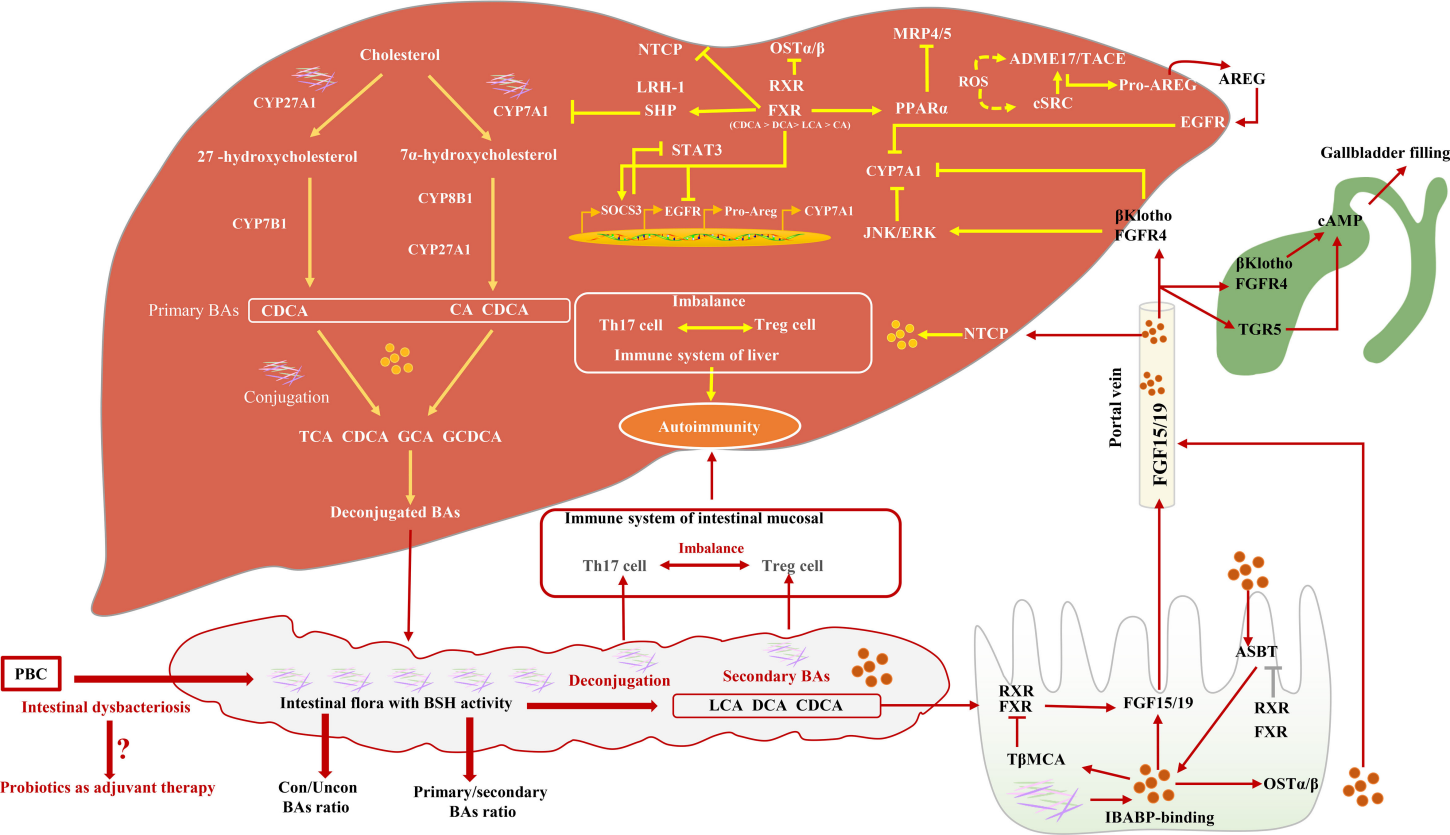
The relationship between intestinal flora and BA synthesis and their possible role in PBC. Intestinal microbial dysbiosis leads to a decrease in BSH and 7α-dehydroxylase activity, subsequently inducing BA pool size and composition changes. Farnesoid X receptor (FXR) activity induces the inhibition of CYP7A1 via activating SHP, the inhibition of Multidrug resistance-associated protein 4/5 (MRP 4/5) via activating Peroxisome proliferator activated receptor alpha (PPARα), and the inhibition of OSTα/β. CYP7A1 is also inhibited by Epidermal growth factor receptor (EGFR) signaling pathways. In the small intestine, BAs, FXR, Apical sodium-dependent bile salt transporter (ASBT), and Ileal bile acid binding protein (IBABP) work together to regulate Fibroblast growth factor 15/19 (FGF15/19), which binds to Fibroblast growth factor receptor 4 (FGFR4) through the portal vein and directly or indirectly inhibits CYP7A1 activity. FGF15/19 induces gallbladder filling through TGR5 or FGFR4 signaling pathways.

The synthesis of BAs involves many reaction steps. The classic BA synthesis pathway is initiated by the 7a-hydroxylation of cholesterol catalyzed by cholesterol 7a-hydroxylase (CYP7A1), which produces about 75% of BA production (mainly including CDCA and CA) (35). The alternative synthesis pathway for BAs is initiated by sterol-27-hydroxylase (CYP27A1), which mainly synthesizes CDCA (32). The proportion of CDCA and CA is mainly regulated by sterol 12a-hydroxylase (CYP8B1) and is not regulated by microbial. However, intestinal flora can regulate the levels of CYP7A1, CYP7B1, and CYP27A1 in the liver (36). Studies have proved that, in the absence of bacteria, the BA pool consists of mainly primary conjugated BAs (37). Specifically, BA deconjugation is carried out by bacteria with bile salt hydrolase (BSH) activity (38), preventing the reabsorption of BAs into the enterohepatic circulation. The deconjugated primary BAs enter the colon and are metabolized by gut microbial 7-dehydroxylation into secondary BAs [Lithocholic acid (LCA) and Deoxycholic acid (DCA)] (32, 39, 40). Gut microbial dysbiosis reduces the activity of BSH and 7α-dehydroxylase, followed by increasing the ratio of conjugated/unconjugated BAs and primary/secondary BAs (41). BAs can affect the composition of intestinal flora through direct antibacterial effects and can also induce antibacterial factors (42) and intestinal protection–related genes (32). Moreover, Amphiregulin (AREG) inhibits the activity of CYP7A1 by targeting the activation of the EGFR signal, preventing toxic BA-induced hepatotoxicity (43). A recent study reported that taking a probiotic (Lactobacillus reuteri) can increase circulating BAs more than two-fold (44). Studies have found the potential role of gut microbes in PBC. However, because of a small number of patients, the difference between gut microbiota and patients with different disease stages and different serum immunological substances has not been studied **Figure 1** (45).

## 3 Small intrahepatic bile duct injury is the main pathological feature of PBC

Biliary epithelial cells (BECs) are the targets of most chronic cholestatic diseases (46). In PBC, continuous damage and senescence of BECs have been observed (47, 48). BECs secrete chemotactic cytokines and inflammatory cytokines during senescence (47, 49, 50) and participate in the induction and recruitment of CD4+ T helper (Th) cells. Toxic BAs can induce mitochondrial dysfunction through oxidative stress in PBC (51) and induce the senescence of BECs through a p38 mitogen-activated protein kinase (p38MAPK)-dependent pathway (52–57). Moreover, the activity of Cyclin-dependent kinase inhibitor p21 (p21WAF1/Cip1) and Cyclin dependent kinase inhibitor 2A (p16INK4) and ataxia telangiectasia-mutated (ATM)/p53/p21WAF1/Cip1 signals in the liver may also induce the senescence of bile duct cells of PBC (48, 58–60). Increasing pieces of evidence suggest that BEC apoptosis may be one of the main mechanisms of the pathogenesis of PBC (61–63). In PBC, emperipolesis of lymphocytes (mainly T lymphocytes) in BECs often be found to be related to inflammation and can reduce the repair of fibrosis (64, 65).

Cholestasis in PBC is related to damage to biliary bicarbonate and a defective biliary bicarbonate “umbrella” on the outer membrane of BECs (46). BECs participate in up to 40% of the bile flow by inducing the active secretion of bicarbonate in the bile (66). By virtue of the favorable Cl^−^ gradient across the plasma membrane of BECs, the activation of Na+-independent anion exchanger 2 (AE2) causes the secretion of bicarbonate in the bile (67). This umbrella is heavily dependent on the AE2 function (68) and prevents hydrophobic BAs from damaging cells (69, 70). The lack of AE2 in the bile duct cells in patients with PBC will cause the cells to be more sensitive to apoptosis induced by cytotoxic hydrophobic Bas (68). There are two separate pathways for bile duct bicarbonate secretion, including cAMP/cystic fibrosis transmembrane conductance regulator (CFTR) signaling and InsP3/Ca^2+^ signaling (71). Type III inositol 1,4,5-trisphosphate receptor (InsP3R3) promotes the secretion of biliary bicarbonate (71, 72). In BECs, pro-inflammatory cytokines enhance the microRNA-506 (miR-506) expression, which can cause overexpression and mislocalization of PDC-E2 (26, 73, 74) in PBC (26, 75–79). There are pieces of evidence that PAMPs that exist in bile can induce the release of chemokines to stimulate the innate immune response and damages the bile ducts (80, 81). Interestingly, mitochondria are present in all cells. However, only BECs are destroyed in PBC (82), which is an important cause inducing autoimmune reactions.

**Figure 2:**
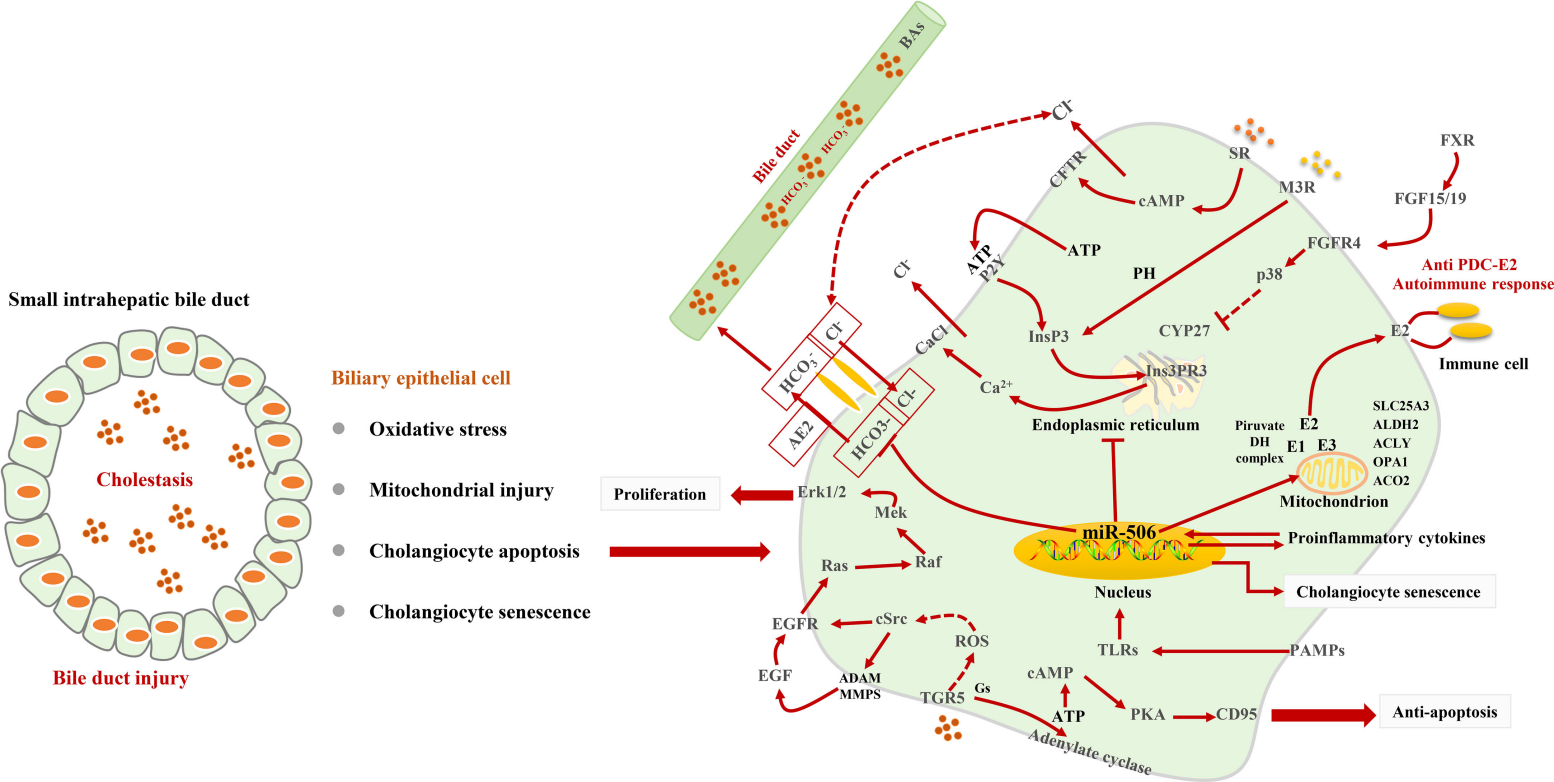
Signaling pathways for BEC injury. Cholestasis induces oxidative stress and mitochondrial injury and leads to BEC senescent and apoptosis. In BECs, TGR5-dependent signaling triggers Cl^−^ secretion, EGFR transactivation, and CD95 to play a role in anti-apoptosis and promote proliferation. The stimulation of M3 muscarinic receptors and P2Y nucleotide receptors induces the release of Ca^2+^ from InsP3Rs. miR-506 targets AE2 to impair biliary bicarbonate secretion and leads to cholestasis. miR-506 can promote the release of PDC-E2.

Multiple BA receptors, including nuclear receptors FXR (83), plasma membrane–bound G protein–coupled receptors (GPCRs) (84), the sphingosine-1-phosphate receptor 2 (S1PR2) (85), and the Takeda G protein–coupled receptor 5 (TGR5) (86), are expressed in BECs and show a variety of biological activities. S1PR2 is only activated by conjugated BAs (86). TGR5 is activated by BAs by increasing reactive oxygen species (ROS) and subsequent EGFR-dependent signals (87) to promote the proliferation of BECs. Ligand binding to TGR5 through coupling to a Gα(s) protein activates adenylate cyclase, increases intracellular cAMP concentrations, and triggers chloride secretion via the CFTR (36, 88–91). BAs can activate FXR and induce the expression of FGF15/19, thereby activating p38 in adjacent BECs and inhibiting the activity of CYP27 (92). Interestingly, in a similar inflammatory environment, compared with other chronic cholestatic liver diseases, the expression of WAF1 and p53 in the BECs in patients with PBC is more significant, which is related to bile duct epithelial cell apoptosis (93) **Figure 2**.

## 4 “Bile acid–inflammation–bile acid” signal axis in PBC

**Figure 3:**
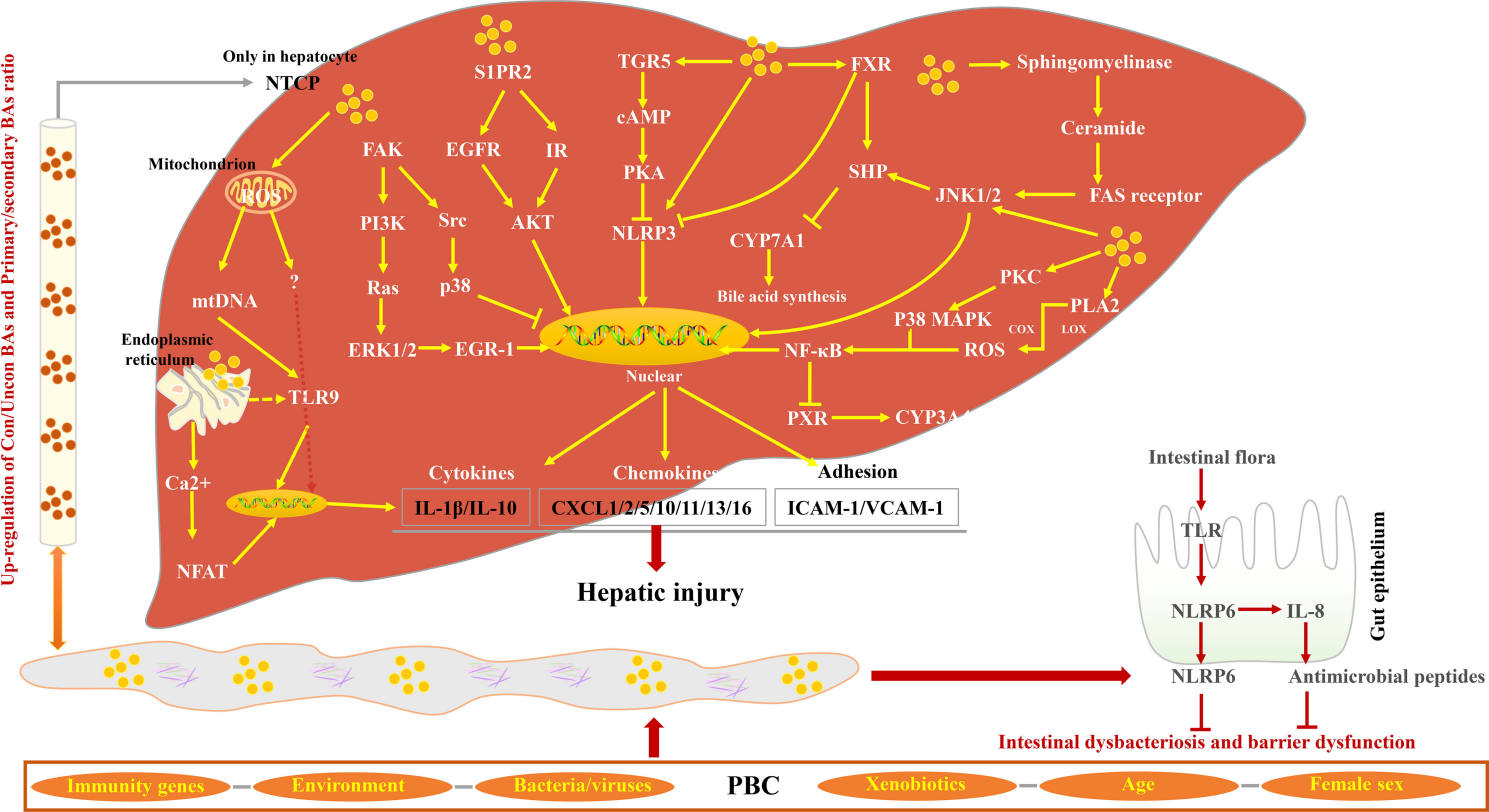
Characteristics of BA-induced inflammatory response in PBC. BAs from enterohepatic circulation activate Focal adhesion kinase (FAK)/EGR-1, FAK/p38, S1PR2/AKT, and sphingomyelinase/Fas cell surface death receptor (FAS) receptor signaling pathways, inducing the release of cytokines, chemokines, and adhesions and subsequently promoting hepatic injury. BAs play an inflammatory effect by promoting the production of ROS and Ca^2+^. In contrast, the ligands of FXR and TGR5 inhibit NLRP3 activity via different signaling pathways. In the small intestine, intestine flora activates TLR, promotes NLRP6 activity, and inhibits intestinal dysbacteriosis and dysfunction.

Studies have shown that the pro-inflammatory effects of hydrophobic BAs mediate liver inflammation through various signal pathways (94–100). Normally, 95% of the BAs in the serum of patients with cholestasis are in conjugated form and are rapidly secreted into the gallbladder once they have been transported into the liver cells by Na(+)/taurocholate cotransporter (Ntcp). In the state of cholestasis, BA efflux transporters are restricted, leading to cholestasis in liver cells (101). NTCP only exists in hepatocytes; therefore, BA-induced inflammatory cytokine production is specific to hepatocytes (102). Excessive BAs in hepatocytes can cause the abnormal expression of cytochrome c and Grp78, leading to the release of mitochondrial DNA, which initiates innate immune response by activating Toll-like receptor 9 (TLR9)-dependent and TLR9-independent signals (103). In hepatocytes, FXR can inhibit NF-κB signaling activity by binding between Nuclear Factor Kappa-beta (NF-κB) and DNA sequences. In addition, the NF-κB p50/p65 heterodimer inhibits FXR-mediated gene [Organic solute transporter alpha/beta (OSTα/β), Bile salt export pump (BSEP), Multidrug resistance-associated protein homologs 2 (MRP2), Multidrug resistance protein-2/3 (MDR2/3), Small heterodimer partner (SHP)]) expression by binding to the FXR promoter in turn (104–107). BAs with high concentration can cause the ubiquitination and phosphorylation of NLR family pyrin domain containing 3 (NLRP3) (108) through the TGR5-cAMP-Protein kinase A (PKA) signal axis and inhibit its activity (109, 110). However, because of the expression restriction of FXR and TGR5 in cholestasis, the activity of NLRP3 cannot be restricted to cause liver inflammation (111). Nuclear factor of activated T cells (NFAT) plays an important role in the inflammatory response in PBC (112–114). Studies suggest that the Ca^2+^–calmodulin–calcineurin–NFAT signaling pathway is involved in BA-induced expression of chemokines in hepatocytes (115), which subsequently recruits neutrophils to mediate the inflammatory response, leading to hepatocyte necrosis (103). Previous studies have confirmed that NFAT signals can interact with innate immune responses through TLR signals (116). The activated TLR9 stimulates Burton’s tyrosine kinase (BTK) and causes phosphorylation and activation of phospholipase Cγ, which, in turn, induces Ca^2+^/NFAT signal (117). However, whether the Ca^2+^/NFAT axis is a downstream signal of TLR9 remains to be discussed (103). NLRP6 inflammasomes in intestinal epithelial cells can inhibit the destruction of the intestinal barrier by inducing Interleukin 18 (IL-18) synthesis and promote the production of antimicrobial peptides and mucus secretion by goblet cells **Figure 3**.

## 5 “Cholestasis–inflammation–liver tissue-specific autoimmunity” signals in PBC

T-cell dysfunction is an important mechanism for PBC (118). The CD4+ cell ratio in the portal area is relatively high. It can develop into two subtypes including T helper type I (Th1) and Th2 cells, respectively, producing corresponding cytokines. Th1 is mainly responsible for cellular immune response (119). The distribution of Th1 is consistent with CD8+ cells in the small bile ducts. CD8+ cells are the main type of lymphocytes that invade BECs in early-stage PBC. However, in the later stage of PBC, the number, proportion, and distribution of immune cells have changed greatly (64, 120). T lymphocytes mediate BEC damage through three pathways: Fas/FasL, T cell receptor (TCR)/major histocompatibility complex (MHC) , and Fas/Fas receptor signaling pathways (63, 64). Targeting nudt1 to inhibit the number and function of CD 103+ T_RM_ cells in the liver has the potential to alleviate immune bile duct injury in patients with PBC (121). Some studies have also found that humoral immune response may also be involved in the inflammatory response in PBC (122, 123), and the CD5–B-cell population may enhance the process of T cells invading BECs (124). The serum and liver tissue cytokine profiles of patients with PBC showed activation and liver recruitment of Th1 and Th17 cells (50).

It has been shown that pro-inflammatory cytokines are involved in the immune response in PBC (125). IL-12 is mainly responsible for Th0 differentiation into Th1 cells. IL-12 can also stimulate the growth and function of T cells. The presence of significant hepatic autoantibodies in IL-12Rβ1–deficient patients suggests that IL-12Rβ1 signaling is closely related to hepatic autoimmunity (126). IL-23 can mediate Th17 differentiation from CD4 T cells and produce IL-6, IL-17, and Transforming growth factor beta (TGF-β) (50, 54). The IL-23– and IL-17–positive monocytes in the portal area in PBC were significantly higher. IL-23 promotes Th0 differentiation into Th17 and induces it to secretion IL-17 (27, 127). Previous studies have proved that IL-6 is essential for Th17 polarization (52, 54). Pro-inflammatory cytokines can activate NK cells and induce the activation of dendritic cells (DCs). DC can further activate T lymphocytes, leading to their differentiation toward the Th1 and Th17 phenotypes. Moreover, it can attack B cells to produce AMA autoantibodies. AMA can recognize the PDC-E2 antigen produced by apoptotic BECs, leading to the formation of antigen–antibody complexes and promoting cell damage (128). IL-17 in PBC not only has a pro-inflammatory effect but may also promote the activation of stellate cells, thereby promoting the occurrence of liver cirrhosis (125, 129). This is also an important factor that PBC progresses from liver fibrosis to cirrhosis. Regulatory T (Treg) cells, as “immune suppressors”, play a protective role in controlling the inflammatory response. Tregs in the damaged bile duct area in the PBC liver tissue are reduced (130). There is increasing evidence that Treg level increases significantly near the inflamed portal area (131). Th17 and Treg cells seem to maintain a delicate balance in liver immune homeostasis (132). It has been found that the main effect of the absence of TGFβ pathway signaling is to downregulate immune regulatory processes and, consequently, upregulate inflammatory processes. In the mouse PBC model, there are extensive differences between the dominant-negative transforming growth factor β receptor II (dnTGFβRII) and normal, wild-type Tregs. Key transcription factors in dnTGFβRII Tregs are downregulated and express an activated pro-inflammatory phenotype (133).

**Figure 4:**
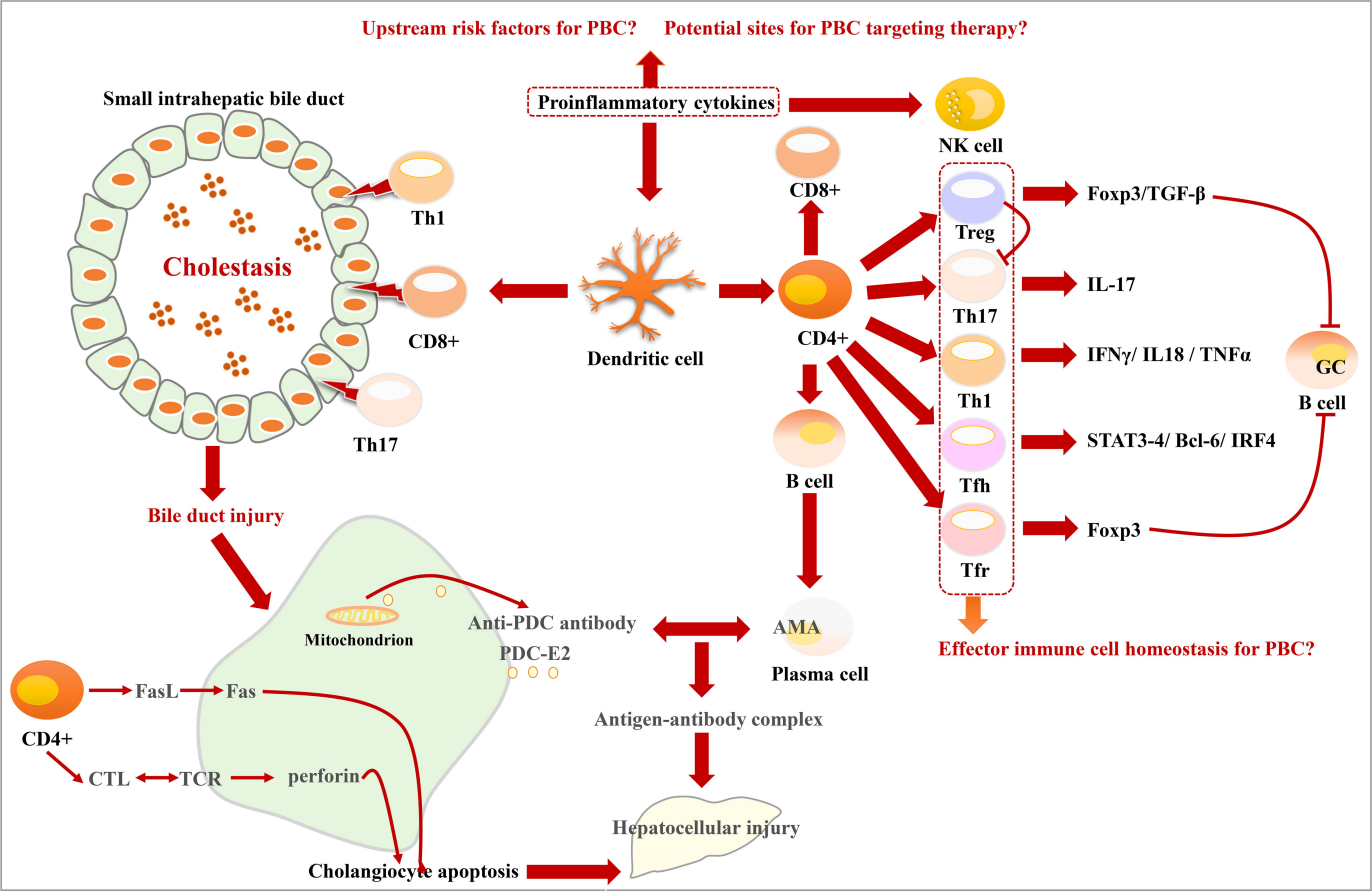
Characteristics of bile duct injury and immune disorder in PBC. DCs activated by proinflammatory cytokines activate T lymphocytes and prompt them to differentiate into Treg, Th17, Th1, Tfh, and Tfr phenotypes. Th1, CD8+, and Th17 cells promote bile duct injury. CD4+ cells induce cholangiocyte apoptosis through cytotoxic T lymphocytes (CTL)/TCR and Fasl/Fas signaling pathways. The mitochondria in damaged cholangiocytes produce PDC-E2 antigen, which can be recognized by AMA, leading to the formation of antigen–antibody complexes.

Studies have found that follicular helper T (Tfh) cells, which are differentiated from CD4+ T cells, can promote B-cell activation, proliferation, affinity maturation, and differentiation (134, 135). They contribute to the B-cell germinal center (GC) response, which is then involved in the development of PBC (132, 136, 137). Tfh cells also express a series of transcription factors, such as Signal transducer and activator of transcription 3/4 (STAT3/4), Transcription factor B-cell lymphoma 6 (Bcl-6), Interferon regulatory factor 4 (IRF4), c-musculoaponeurotic fibrosarcoma (cMaf), and show a similar relationship with Th effector subsets (138). Another study also found that the survival, proliferation, and differentiation of GC B cells and Tfh cells are reciprocally dependent on each other (138). Tfr cells can express Foxp3 and suppress the same function as Treg cells (139, 140). Intrahepatic Treg cells and Tfr cells have inadequate inhibition of inflammatory and autoimmune responses (141). In GWAS studies, genetic factors have been found to play a key role in the development of primary cholangitis (PBC). Relatives of patients with PBC had a significantly higher risk of developing the disease, and, even among second - and third-degree relatives, the risk was significantly increased (19, 142). Because of the insufficient sample size of patients with PBC, GWAS has not revealed the key pathological mechanism of PBC. A new CD103+ CD69+ CD8+ T cell has recently been found to invade BEC, and the E-cadherin expressed by this type of cell accelerates its invasive ability **Figure 4**.

## 6 Natural small molecules have great potential in the treatment of PBC

Currently, there are a limited number of drugs that have been approved for clinical use in the treatment of PBC. Natural small molecules are an essential area of research for candidate drugs for PBC treatment. FXR agonists have been found to be a promising direction for PBC-targeted drug research. The approved FXR agonist OCA has shown outstanding therapeutic efficacy in the clinical treatment of PBC (143). It is noteworthy that multi-acid receptor agonists have more tremendous therapeutic potential for PBC (144). On this basis, we reviewed the research progress of laboratory and clinical research on FXR agonists (**Table 1**).

**Table 1:** Natural small molecules that activate nuclear receptors.

No.	Natural small molecule	Component source	Targeted targets	Research stage
1	Geniposidic acid (145)	Fructus Gardeniae	**Fxr↑**, Bsep↑, Mrp2↑, Sirt1**↑**, MRP2**↑**, NTCP**↑**, ASBT**↑**, and IBABP**↑**	Preclinical study
2	Resveratrol (146)	Polygonum Cuspidatum	**Fxr↑,** Cyp7a1↓, Cyp8b1↓, Ostβ↓, Mrp3↓, Cyp27a1↓, and Shp**↑**	Preclinical study
3	Alisol B 23-acetate (147–149)	Alisma orientale	**Fxr↑, PXR↑,** Cyp7a1↓, Cyp8b1↓, Sult2a1**↑,** Bsep**↑**, Mrp2**↑**, Ntcp↓Shp**↑,** Fgf15↑, and Mdr2**↑**	Preclinical study
4	Yangonin (150, 151)	Kava	**Fxr↑,** Bsep**↑**, Mrp2**↑**, Ntcp↓, Cyp7a1↓, Cyp8b1↓, and Sult2a1**↑**	Preclinical study
5	Calycosin (152)	Radix Astragali	**Fxr↑,** FoxM1B**↑**, Shp**↑**, and Bsep**↑**	Preclinical study
6	Curcumin (153)	Curcuma aromatica Salisb	**Fxr↑,** Bsep**↑**, Mrp4↓, Ostβ↓, Cyp7a1↓, Cyp8b1↓, and Mrp4**↑**	Preclinical study
7	Picroside II (154)	Picrorhiza scrophulariiflora Pennell	**Fxr↑,** Bsep**↑**, Sult2a1**↑,** Cyp7a1↓, and Cyp8b1↓	Preclinical study
8	Emodin (155)	Rhubarb	**Fxr↑** and Bsep**↑**	Preclinical study
9	Auraptene (156)	Grapefruit	**Fxr↑,** Bsep**↑**, Mrp2, Ntcp↓, Cyp7a1↓, Cyp8b1↓, and Sult2a1**↑**	Preclinical study
10	Corilagin (157)	Phyllanthus urinaria	**FXR↑,** SHP1**↑**, SHP2**↑**, UGT2B4**↑**, BSEP**↑**, MRP2**↑** and SULT2A1**↑,** CYP7B1↓, and NTCP↓	Preclinical study

↓ represents downregulation of gene expression, ↑ represents up-regulation of gene expression.

## 7 Conclusion

By summarizing the mechanism of PBC in BA cytotoxicity, the correlation characteristics of inflammation with BAs and immune response, and the main targets of hepatocyte necrosis and senescence and apoptosis of BEC, we have a generally in-depth understanding of the pathogenesis of PBC. Immune pathogenesis has a special relationship with the development of PBC. The immune response involves both innate immune response and humoral immune response that directly damage liver cells and BECs. However, the clinical effects of existing classic immunosuppressants and clinical trials of new biological agents modifying the immune system have been disappointing (158–160). Therefore, it is necessary to rethink the role of the immune mechanism in the pathogenesis of PBC. Limiting immune response to “downstream pathogenic factors” cannot achieve the desired efficacy in the treatment of PBC. Therefore, it is necessary to improve the “upstream pathogenic factors” of PBC, such as BA or inflammation, while suppressing immunity. This requires the new functions of new potential therapeutic drugs for PBC. It is worth noting that most patients with PBC have AMA autoantibodies. However, there are still a few patients with PBC who cannot be detected with AMA autoantibodies, but they do have other anti-nuclear antibodies. This suggests that the current research on the immune mechanism is flawed, and the key immune pathogenesis still needs further research and discovery.

As a possible “upstream factor” in the pathogenesis of PBC, BAs also show metabolic abnormalities in viral hepatitis, alcoholic or non-alcoholic steatohepatitis, drug-induced liver injury, and intrahepatic cholestasis of pregnancy, but no AMA autoantibodies appear as in PBC. This distinction suggests whether the relationship between BA metabolism disorder and autoimmune response is mediated by a “third party” in PBC, which requires further study. BAs are synthesized in hepatocytes, excreted into the small intestine through the gallbladder, and absorbed by the liver through blood circulation. Various cells throughout the enterohepatic circulation are exposed to BAs. However, in PBC, only the mitochondria in BECs are damaged. The destruction of mitochondria in BECs is also an important cause of autoimmune reactions. Therefore, we point out that BA-mediated damage to mitochondria in BECs is likely to be mediated by specific components of BECs. This is also an essential step in finding a PBC-targeted therapy strategy. On the basis of the existing research methods and technologies, some new high-throughput and high-sensitivity bioinformatics analysis technologies, such as GWAS, need to be used to reveal the targeting mechanism of PBC.

## Author contributions

YZ generated the idea for the review and wrote the manuscript. SW, LC, XZ, and XM designed the figures corrected the manuscript. All authors contributed to the article and approved the submitted version.
